# The Formation Mechanisms of np-Fe in Lunar Regolith: A Review

**DOI:** 10.3390/ma17235866

**Published:** 2024-11-29

**Authors:** Mingchao Xiong, Yanxue Wu, Wenqing Yao, Zilei Chen, Yingying Yu, Xia Li, Pan Yan, Xiongyao Li, Xiaojia Zeng

**Affiliations:** 1 School of Environmental Science and Engineering, Guangdong University of Technology, Guangzhou 510006, China; xmingchao2022@163.com (M.X.); czlfugace@163.com (Z.C.); yyy011019@163.com (Y.Y.); 2 Analysis and Test Center, Guangdong University of Technology, Guangzhou 510006, China; lix0061@163.com; 3 Department of Chemistry, Tsinghua University, Beijing 100084, China; 4 Planetary Environmental and Astrobiological Research Laboratory, School of Atmospheric Sciences, Sun Yat-sen University, Zhuhai 519082, China; yanp27@mail2.sysu.edu.cn; 5 Center for Lunar and Planetary Sciences, Institute of Geochemistry, Chinese Academy of Sciences, Guiyang 550081, China; lixiongyao@vip.skleg.cn

**Keywords:** np-Fe particles, Chang’E-5′s lunar soil, in situ experiments

## Abstract

Nanophase iron (np-Fe) is widely distributed on the surface of lunar soil particles, forming as a result of space weathering. These np-Fe particles contribute to the reddening and darkening of the visible to near-infrared spectra of weathered lunar material and serve as critical indicators for assessing the maturity of lunar soil. (1) This article reviews the proposed formation mechanisms of np-Fe particles from studies of Apollo and Luna soils, including the thermal reduction of iron melts, vapor deposition caused by micrometeorite impacts, and hydrogen reduction due to solar wind exposure. (2) Additionally, recent findings from the analysis of Chang’E-5 lunar soil are highlighted, revealing new mechanisms such as sub-solidus decomposition of olivine, impact-driven disproportionation, and FeO eutectic reactions. (3) Experimental studies simulating space weathering through laser and ion irradiation are also discussed and compared. Despite extensive research, a definitive understanding of np-Fe particle formation remains elusive. Previous lunar soil samples have been collected from the near side of the Moon. This year, the Chang’E-6 mission has successfully returned the first-ever lunar soil samples from the far side. These samples are expected to exhibit unique space weathering characteristics, providing new insights into the formation mechanisms of np-Fe in lunar soil.

## 1. Introduction

Nanophase iron (np-Fe) in lunar soil is a product of space weathering, found in the amorphous rims and agglutinates of lunar soil particles. In the solar system, the Moon and other airless bodies are continuously exposed to solar wind sputtering and injection (solar wind is a magnetized plasma flow ejected from the Sun, consisting of oppositely charged particles, such as ions and electrons) and meteoroid and micrometeoroid impacts, as well as cosmic ray radiation [1]. The cumulative effects of these processes are collectively referred to as “space weathering” [2,3]. As a result of space weathering, the surface of the Moon undergoes significant physical and chemical changes, leading to alterations in its mineral composition. High-energy ions from solar wind, solar flares, and cosmic rays are implanted into lunar soil particles, causing radiation damage and resulting in the formation of amorphous rims (amorphous rims are noncrystalline and exhibit evidence of the preferential sputtering of cations) and splatter agglutinates [4]. Additionally, meteoroid and micrometeoroid impacts fragment lunar rocks into smaller particles, producing an abundance of impact glasses that constitute approximately 40–75% of the volume [5]. Concurrently, a vapor deposition layer rich in np-Fe particles develops on the surface of lunar soil.

Following the return of the first batch of lunar soil samples from the Apollo 11 mission, researchers observed notable differences in the reflectance spectra between the lunar soil samples and the rock samples collected from the same site. Initially, these spectral differences were attributed to the presence of glass in the lunar soil, although this conclusion was not confirmed at the time [6,7]. It was only after the introduction of transmission electron microscopy (TEM) in lunar soil studies that researchers identified substantial np-Fe particles in the amorphous rims of lunar soil grains. Additionally, the elemental iron content in lunar soil was found to be approximately ten times higher than that in the parent rock [8,9]. These findings led to the association of np-Fe particles with the observed spectral variations. Reflectance spectroscopy serves as a powerful tool for determining the mineral composition of planetary surfaces, but the presence of np-Fe particles may obscure the spectral signatures of certain minerals [10,11]. Further studies indicated that np-Fe particles of varying sizes and quantities have different effects on the reflectance spectra of airless bodies. Specifically, nanoparticles smaller than 5 nm induce a reddening of spectral reflectance, while those around 10 nm result in a decreased total reflectance. In contrast, larger np-Fe particles (>40 nm) may darken the spectra across the entire wavelength range but do not cause reddening [11,12].

In earlier studies, np-Fe particles in lunar soil were mainly reported to consist of elemental iron [3]. This assumption arose from the reducing conditions on the lunar surface and ferromagnetic resonance measurements of the soil [13]. Later research demonstrated that np-Fe particles could exist in different oxidation states [4,14,15]. However, the spatial and spectral limitations of most transmission electron microscopes (TEMs) at that time made it difficult to directly measure the oxidation states of iron in these nanoparticles, preventing conclusive evidence of Fe^2+^ and Fe^3+^. With recent advances in electron optics, direct atomic-resolution imaging and spectroscopy have become possible. Thompson et al. used electron energy-loss spectroscopy (EELS) and Cs-corrected TEM to study lunar soil particles at different maturity levels, showing that the oxidation state of np-Fe particles increases as the soil matures [16]. Immature soils predominantly contain metallic Fe^0^ nanoparticles; submature soils contain both Fe^0^ and Fe^2+^; and mature soils have Fe^2+^ and Fe^3+^. In submature samples, np-Fe particles were found to have oxidized shells, likely due to the diffusion of oxygen atoms from the surrounding oxygen-rich matrix to the surface of the particles. Additionally, changes in the oxidation state of np-Fe particles significantly influence the optical properties of lunar soil. As the oxidation state increases, the spectral slope of mature lunar soil becomes steeper compared to the measured soil [16]. Therefore, when assessing the impact of np-Fe particles on the reflectance spectra of lunar soil, it is essential to consider the distribution of their oxidation states.

There are generally two sizes of np-Fe particles: those smaller than 15 nm and those ranging from 20 to 1000 nm, with larger np-Fe particles mainly found in agglutinates [4]. Lunar soil experiences varying degrees of modification due to space weathering, depending on its location on the Moon. To standardize assessment criteria, researchers introduced the concepts of exposure age and soil maturity. Exposure age quantitatively measures the duration that rocks or soil particles have been exposed to space by analyzing accumulated products like solar wind inert gases or cosmic ray tracks [17]. Studies comparing lunar soil samples with different exposure ages found that the number of np-Fe particles increases with longer exposure times. Consequently, Morris introduced the concept of soil maturity and developed a simple, non-destructive method to determine it: by measuring electron spin resonance (ESR) intensity and calculating the Is/FeO ratio, which is the relative concentration of np-Fe to total iron. This ratio indicates the surface exposure time of lunar soil and classifies it into three categories: immature (0.0 ≤ Is/FeO ≤ 29.0), submature (30.0 ≤ Is/FeO ≤ 59.0), and mature (Is/FeO ≥ 60.0) soil [18].

The presence of np-Fe particles not only influences remote sensing spectra but also holds valuable information about the evolution of lunar soil. Since np-Fe particles are products of space weathering, studying them allows researchers to infer the processes the Moon has undergone. Additionally, np-Fe particles alter the sintering characteristics and metallurgical properties of lunar soil, providing essential insights for constructing lunar bases [19,20]. Space-weathered samples produce 5 to 100 times higher concentrations of oxidized OH* in deionized water compared to non-space-weathered simulated lunar soil, which is linked to metallic iron. This suggests that inhaling space-weathered lunar soil could cause significant oxidative damage to human organs [21]. Both the U.S.A and China have initiated crewed lunar missions, making the potential health risks of np-Fe particles for astronauts a critical concern [22,23].

Moreover, np-Fe particles are also present on other airless bodies similar to the Moon, and the findings from np-Fe research can be applied to these bodies as well. Examples include Mercury, Ryugu, Vesta, Itokawa, and Bennu, which hold great significance for spacecraft exploration of new worlds such as Phobos, Deimos, and the outer solar system’s satellites [24]. Space weathering is primarily driven by solar wind and micrometeoroid impacts, with cosmic rays contributing to a lesser extent. To better understand the formation of np-Fe, researchers have conducted numerous simulated experiments that replicate solar wind and micrometeoroid impacts. For example, high-energy ions like H^+^, He^+^, and Ar^+^ have been used to simulate solar wind irradiation [25,26,27,28]. Additionally, pulse laser irradiation with varying wavelengths, durations, and frequencies has been employed to mimic micrometeoroid impacts [29,30,31,32], along with heating simulations to simulate micrometeoroid impacts [33,34]. Despite the different proposed explanations for np-Fe particle formation, no definitive conclusion has been reached due to the limited availability of lunar soil samples.

**Figure 1:**
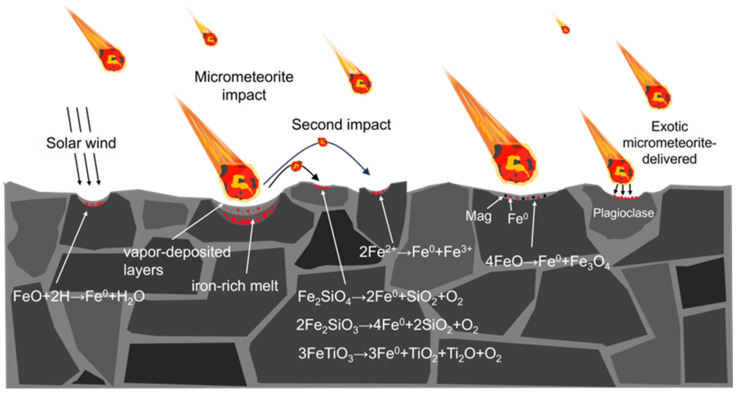
Formation mechanism of np-Fe particles in lunar regolith.

On 17 December 2020, after a 44-year gap, the Chang’E-5 mission successfully returned with new lunar soil samples. These samples are the youngest and from the highest latitude among all collected lunar samples [35,36]. Studies have shown that the Chang’E-5 lunar regolith mainly consists of locally sourced mare basalt formed from a single lava flow, with only trace amounts of exogenous material [37]. Through the analysis of these samples, researchers have proposed new mechanisms for np-Fe particle formation, including disproportionation, decomposition, and eutectic reactions (Figure 1). Over the past 44 years, there have been significant advancements in microregion characterization technologies, with in situ experiments now widely used in materials science. This method consumes minimal sample material and allows the real-time observation of changes using TEM during experiments. This review summarizes the various formation mechanisms of np-Fe particles identified in Apollo, Luna, and Chang’e 5 lunar soil, highlights the challenges in distinguishing these mechanisms, and proposes future research directions. We believe in situ experiments are the future of simulating space weathering, and our upcoming research will focus on this approach to determine the formation mechanism of np-Fe particles.

## 2. Discussion of the Formation Mechanisms of np-Fe

**Figure 2:**
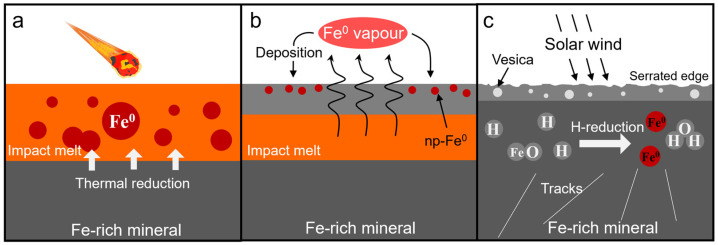
Formation mechanisms of three traditional np-Fe particle types: (**a**) in situ thermal reduction caused by micrometeorite impacts; (**b**) vapor deposition caused by micrometeorite impacts; (**c**) H-reduction by solar wind injection.

Since the 1970s, when np-Fe was first detected using magnetic and electron spin resonance (ESR) techniques, several hypotheses have been proposed to explain its formation. Currently, three primary mechanisms, based on the study of regolith samples from the Apollo and Luna missions, are widely accepted. The first mechanism involves the in situ thermal reduction of lunar soil during the melting process caused by micrometeorite impacts (Figure 2a) [6,38,39]. The second suggests that np-Fe forms through vapor deposition resulting from solar wind sputtering or micrometeorite impacts (Figure 2b) [40,41]. The third mechanism proposes that np-Fe is produced by the reduction of oxidized iron through solar wind-implanted hydrogen in the lunar regolith (Figure 2c) [42]. These mechanisms highlight the roles of micrometeorite impacts and solar wind in np-Fe formation. However, the hydrogen reduction hypothesis has been questioned, as many simulation experiments indicate that np-Fe formation does not necessarily require hydrogen [43]. In contrast, the in situ thermal reduction and vapor deposition hypotheses have gained some support from experimental simulations. Despite this, a definitive formation mechanism has yet to be established from the returned lunar samples [44,45]. It is generally believed that small np-Fe particles in the lunar soil granular layer originate from solar wind reduction or vapor deposition, whereas larger np-Fe particles in the amorphous layer are formed through thermal reduction caused by micrometeorite impacts.

### 2.1. Thermal Reduction Induced by Micrometeorite Impacts

In the returned lunar soil samples, numerous np-Fe particles are observed by TEM within the melted glass at the bottom of the lunar regolith, a region inaccessible to hydrogen and helium. This implies that the formation of these np-Fe particles is not driven by the solar wind. Numerous researchers have replicated this phenomenon through simulation experiments [25,31,38,46]. Thompson et al. (2017), using TEM real-time observations, documented the formation of np-Fe particles under heating conditions and their gradual fusion and growth with extended heating [46]. Based on this, some researchers have proposed the in situ thermal reduction hypothesis, suggesting that micrometeorite impacts heat and melt iron-rich minerals, where ferrous iron is thermally reduced by oxygen ions (O^+^) to form metallic iron. This iron, immiscible with the silicate melt, separates as liquid metallic droplets. These droplets, rich in iron, disperse from the silicate melt, and early-separated droplets may coalesce into larger ones to minimize surface energy, resulting in np-Fe particles of varying sizes [39,47]. The chemical reaction involved can be expressed as

2FeO → 2Fe^0^ ↓ + O_2_ ↑ (1)

In both natural lunar soil and simulated experiments, np-Fe particles tend to form chain-like or reticular patterns, which is believed to result from the preferential arrangement and concentration of iron-rich melt in the high-temperature regions of silicate melt [39]. Due to the high interfacial tension between the iron-rich melt and the surrounding silicate, immiscibility and rapid cooling lead to the formation of spherical iron-rich grains [47,48]. This process also produces numerous glass spheres, resulting in glass-encased np-Fe particles [49]. Although hydrogen is not involved in this np-Fe formation mechanism, it is consistent with many observations of lunar regolith. Keller’s comprehensive study of natural lunar soil particles found that np-Fe located at the edges of lunar soil particles is mainly produced by impact processes rather than by solar wind [50]. Additionally, Li et al. (2022) observed that the major cationic components in the residue of a microcrater formed by low-velocity micrometeorite impact were Ca, Al, and Si, similar to plagioclase found on the lunar surface [51]. Inside the crater, np-Fe particles were present, but they were almost absent in the surface residue. The lack of iron in the surface residue, along with the absence of sulfur and nickel in the internal np-Fe, suggests that the np-Fe formed through in situ reduction triggered by the impact, rather than by evaporative deposition. Moreover, numerous pulsed laser simulations of micrometeorite impacts have demonstrated that, even in the absence of reducing hydrogen, various irradiated minerals can form melt layers containing np-Fe particles [38,52].

### 2.2. Vapor Depositions Induced by Micrometeorite Impacts

**Figure 3:**
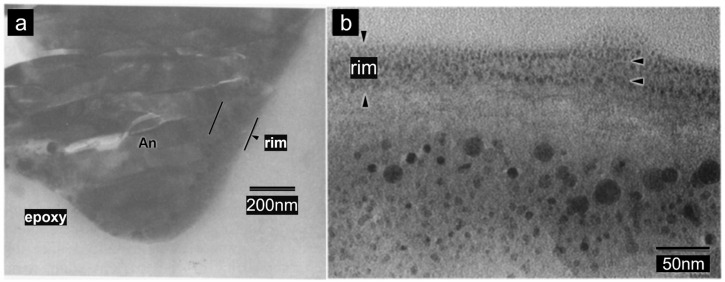
(**a**) Bright-field TEM image of a thick amorphous rim on an anorthite grain from lunar soil sample 61181. The dark inclusions in the rim are mostly Fe metal with a few FeS grains [8]. (**b**) High-magnification TEM image of vapor-deposited amorphous coating on melt glass. The layering of np-Fe particles within the rim indicate multiple episodes of vapor deposition [3].

Np-Fe is found not only in impact glasses but also in vapor-deposited layers on the surfaces of mineral grains (Figure 3a,b) [53,54]. These vapor-deposited layers coat the lunar soil particles, and researchers using scanning electron microscopy (SEM) and transmission electron microscopy (TEM) have observed that they lack a crystalline structure. This suggests that these amorphous rims may also form due to solar wind irradiation [3,55]. However, chemical analyses of these rim materials reveal that their composition differs from the underlying minerals, indicating they result from vapor deposition after micrometeorite impacts. The contribution of solar wind to the amorphization process appears minimal [8].

In the vapor deposition layer, there is a notable depletion of refractory elements like Al, Ca, and Ti, while volatile elements such as Si, Fe, and S are enriched [56]. Some vapor-deposited layers exhibit stratification, indicating multiple deposition events on weathered grain surfaces. A new mechanism has been proposed to explain the origin of np-Fe particles. Under micrometeorite impacts, iron oxides in lunar minerals are heated and decomposed, producing vapor-phase iron [2,41] that escapes to the surface. Through physical processes, the iron is reduced by the selective loss of oxygen during vapor deposition. The iron particles then aggregate into nanoscale iron spheres, which cool and solidify. The proposed reaction can be represented as follows:

2FeO → 2Fe^0^ ↓ + O_2_ ↑ (2)

The origin of np-Fe has been debated, primarily between two mechanisms: H-reduction by the solar wind and vapor deposition from micrometeoroid impacts. These processes would result in different iron isotope fractionation effects in np-Fe. In the first case, where solar wind reduction occurs directly on the lunar soil surface, no significant iron isotope fractionation is expected. In contrast, vapor deposition from impacts is associated with a kinetic isotope effect, where lighter isotopes escape more readily, enriching heavy isotopes in the deposited material compared to the parent rock and weathered layer [52]. This makes iron isotope analysis a key tool in determining the origin of np-Fe. Early research measuring the ^56^Fe/^54^Fe isotope ratio (δ^56^Fe) in thin layers of lunar soil detected fractionation effects up to 0.3 per mil (0.03 ± 0.05‰) relative to the parent rock [57,58], which was linked to np-Fe as δ^56^Fe increased with the maturity index Is/FeO. Similarly, Wang et al. (2012) found that np-Fe on weathered plagioclase feldspar showed heavy iron isotope enrichment (δ^56^Fe up to 0.71‰) compared to bulk plagioclase and other lunar materials (δ^56^Fe around 0.3‰) [59]. Plagioclase, being a nominally Fe-free mineral, indicates that this np-Fe could not have been formed through solar wind reduction. While iron meteorites exhibit higher δ^56^Fe values, they remain too low to account for the δ^56^Fe = 0.71‰ end-member observed here, suggesting that this np-Fe cannot result from contamination by meteorite material [60]. As previously mentioned, vapor deposition contributes to the enrichment of δ^56^Fe, which plays a key role in the formation of np-Fe. Thermal escape models support this, showing that lighter iron isotopes preferentially escape into space during vapor generation from solar wind sputtering and micrometeoroid impacts, explaining the np-Fe’s isotopic composition. Overall, isotopic studies strongly indicate a vapor deposition origin for np-Fe in lunar regolith [59].

Vapor deposition not only facilitates the formation of np-Fe particles but also influences the creation of other minerals. During impacts, common vapor species include Fe, SiO, O_2_, and O, with oxygen being the most volatile. This process leaves iron in its metallic state due to the absence of oxidation. Recently, Guo et al. (2023) identified digenite (CuS) for the first time in lunar regolith samples returned by the Chang’E-5 mission, found adhering to a micrometer-sized iron particle [61]. Compositional analysis revealed that this mineral also formed through vapor deposition. Furthermore, Li et al. (2024) reported the first discovery of native copper and FeCo alloy within a lunar basaltic clast from the Chang’E-5 mission [62].

### 2.3. H-Reduction Induced by Solar Wind Irradiation

The solar wind, comprising over 99% hydrogen and helium ions, continually bombards the lunar surface, leading to accumulation within the lunar regolith [63]. Prolonged exposure to the solar wind disrupts the crystalline structure of the lunar soil’s surface layer, resulting in the formation of an amorphous layer [64]. Some researchers propose that np-Fe particles form through the reduction of iron oxides in lunar soil, driven by hydrogen reduction induced by solar wind irradiation [42,65,66]. Following ion irradiation, surface materials, particularly oxygen atoms, preferentially sputter from lattice positions, creating a reducing environment. The remaining iron particles in the surface material are subsequently reduced to np-Fe. If micrometeoroid impacts occur during this process, they can heat the silicate minerals, leading to glass formation and a more intense reduction process. This results in the production of relatively large metallic iron spheres (approximately 20 nm) within the glass matrix [42,67,68,69]. The reaction can be summarized by the following equation:

FeO + 2H → Fe^0^ + H_2_O ↑ (3)

Traces of solar wind can be preserved on certain lunar soil particles. For instance, jagged edges may develop on particle surfaces, ion implantation tracks can be detected within the particles, and small vesicles may form on np-Fe particle surfaces, allowing elements like hydrogen and helium to be stored [70,71]. Cymes et al. (2021) reported helium K-edge peaks in the “pockets” of two vesicular np-Fe particles through an electron energy-loss spectroscopy (EELS) analysis of Apollo samples [72]. Nanoscale observations indicate that np-Fe particles within impact-generated lunar glass contain high concentrations of helium-3 from space weathering, suggesting they may serve as significant helium reservoirs [73].

With advancements in research and observational techniques, our understanding of the Moon has deepened, leading many scholars to question the H-reduction hypothesis. First, solar wind ions are implanted into weathered grains at only a few hundred angstroms per second. Given their small size, hydrogen ions can easily diffuse out of these particles with minimal temperature changes, leaving insufficient time to react with iron in the minerals [41]. Second, forming np-Fe particles requires not only the reduction of iron ions to atomic states but also their aggregation into spherical structures, which the hypothesis does not fully explain. Additionally, no H_2_O, a byproduct of the H-reduction reaction, has been found in the glass encapsulating np-Fe particles. In situ spectral observations reveal that the average hydroxyl content in lunar soil at the Chang’E-5 landing site is 28.5 ppm, suggesting a minimal contribution from solar wind injection [74]. Notably, Sasaki et al. (2001) conducted pulsed laser irradiation experiments on olivine samples in a vacuum, without implanted hydrogen, and observed np-Fe particles in the amorphous vapor-deposited layer on olivine edges [43]. This finding aligns with the np-Fe found in the regolith differentiation layer, indicating that solar wind-injected hydrogen is not essential for np-Fe particle formation. In addition, Thompson et al. (2017) simulated micrometeorite impacts on lunar soil particles using in situ heating experiments, observing the real-time formation of np-Fe particles inside the amorphous layer via TEM [46]. These experiments were conducted under vacuum conditions, minimizing the presence of atmospheric gases that could interact with the sample during heating. Since H^+^ ions from the solar wind cannot reach this depth, the formation of np-Fe particles at the bottom of the amorphous layer does not require the presence of H^+^.

While solar wind ion irradiation may not reduce iron oxides, it has been shown to reduce troilite (FeS) to metallic iron on asteroids [75]. Remote sensing of the S-type asteroid 433 Eros has revealed substantial sulfur depletion in its weathered surface layer, attributed to the space weathering of iron sulfides [76,77]. Laboratory simulations demonstrate that solar wind preferentially removes sulfur, enriching the mineral surface with iron. Notably, sulfur depletion rates from H^+^ and He^+^ are similar, and both ion types induce nanoscale surface roughening [25,71]. Given that iron sulfide is a common mineral throughout the solar system and widely distributed on the Moon, it is likely that solar wind-induced sulfur depletion and iron reduction also occur on the lunar surface.

### 2.4. The np-Fe on Other Airless Bodies

In the solar system, airless bodies like Mercury, Eros, Itokawa, Gaspra, Ida, Phobos, and Deimos undergo space weathering processes similar to those on the Moon. Spacecraft missions, including MESSENGER, NEAR, and DAWN, have enabled spectral observations and sample returns from these bodies, allowing researchers to analyze and compare the morphology, abundance, and primary formation mechanisms of np-Fe particles across different airless celestial bodies [78,79,80].

The impact of space weathering on each celestial body is shaped by factors such as its solar system position, which influences impact velocity, radiation exposure, and temperature, as well as its composition, physical properties, and surface exposure history [7,45]. Although Mercury has a weak magnetic field, it cannot entirely shield itself from solar wind erosion. With a stronger gravitational field than the Moon, Mercury experiences higher impact velocities and flux, leading to a more abundant vapor deposition layer. It is thought that most iron on Mercury, from both internal and external sources, has been reduced by vapor deposition [7]. Frequent impacts on Mercury lead to repeated surface melting, and through Ostwald ripening, smaller np-Fe particles coalesce into larger ones [11,81]. Models predict that the ratio of larger to smaller np-Fe particles on Mercury is about three times that on the Moon [82]. Additionally, simulation experiments indicate that Mercury’s surface temperature can transform magnesium olivine into np-Fe within hours and sm-Fe within a few million years, altering its spectral characteristics [83].

The asteroid Gaspra does not exhibit measurable space weathering products like the np-Fe particles found on the Moon. This may be due to two main factors: first, Gaspra’s lower solar wind ion flux and fewer micrometeorite impacts contribute to weaker space weathering, which makes the formation of np-Fe particles challenging; second, Gaspra has a limited presence of iron and sulfides, essential elements for np-Fe particle formation, reducing the likelihood of their production at the source [84]. Conversely, the asteroid Ida, with its water ice, hydrated materials, and volatile-rich composition, also makes the formation of np-Fe particles similar to those on the Moon unlikely [7].

The asteroid Itokawa is the only extraterrestrial body, aside from the Moon, from which samples have been collected. The Hayabusa mission returned hundreds of particles from the Muses Sea region, representing the weathered surface of Itokawa [79,85]. Compared to the Moon, impact velocities on Itokawa’s surface are much lower, averaging around 5 km/s, which generally results in fragmentation rather than melting or vaporization, thus limiting np-Fe formation from vapor deposition [86]. The vapor-deposited layer on Itokawa particles is thin (5–15 nm), containing nanophase opaque material (npOpq) with particle sizes around 2 nm and sulfur, in addition to iron. Some sulfur-free np-Fe particles are embedded deep within iron-bearing silicates (up to 60 nm), where Fe^2+^ is reduced in situ. These np-Fe particles are found at the edges of amorphous layers created by solar wind exposure, a process similarly observed in lunar soil, especially within olivine [17].

## 3. New Views Revealed by Chang’E-5 Lunar Samples

With the return of the Chang’E-5 mission in 2020, new lunar soil samples became available for study. The mission landed on a lunar mare basalt and collected 1731 g of soil, including samples scooped a few centimeters below the surface and core samples drilled approximately one meter into the lunar regolith [87]. The geological age of these samples is estimated at around 2.0 billion years, classifying them as relatively young lunar soil [36]. Magnetic and spectral analyses identify Chang’E-5 soil as among the least mature lunar soils, with a metallic iron abundance of 0.42 % and an Is/FeO value of 14 [88]. Long-term space weathering typically increases soil maturity and np-Fe content but can obscure the initial characteristics of lunar soil formation, complicating the study of np-Fe sources [89]. The limited space exposure of the CE-5 soil suggests mild space weathering effects, offering a rare glimpse into the early stages of np-Fe formation.

**Figure 4:**
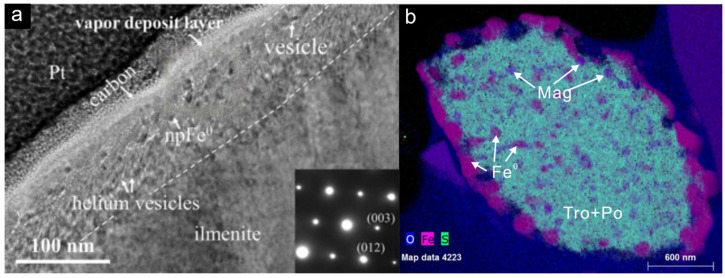
(**a**) TEM image of ilmenite surface. A vapor deposit layer is present beneath the coatings and shows an island-like morphology. Below the vapor deposit, the solar wind-damaged zone (~80 nm) contains irregular vesicles coexisting with np-Fe particles in the upper part and parallel helium vesicles in the np-Fe-free lower part [27]. (**b**) Quantitative energy-dispersive X-Ray spectroscopy mapping of iron sulfide grains shows the coexistence of magnetite and np-Fe particles [99].

Researchers have identified abundant np-Fe particles in the Chang’E-5 samples, alongside micrometeoroid craters, impact-induced splashes, nanoscale mineral fragments, and dispersed metallic microspheres. These features indicate typical space weathering effects, such as visible and near-infrared spectral darkening and reddening (Figure 4a) [37,90,91]. The Fe^0^ content (>17 wt%) was derived through in situ and laboratory VNIR measurements of the returned samples [92]. The study of Chang’E-5 lunar soil revealed that np-Fe particles vary in size and depth across different host minerals. TEM observations show that np-Fe particles are larger in low-Ca, high-Fe pyroxene lamellae, with an average diameter of 4.6 ± 0.7 nm, compared to those in high-Ca, low-Fe pyroxene lamellae, which have an average diameter of 3.5 ± 0.8 nm. The largest np-Fe particles are found on the jagged surfaces of iron sulfide, ranging from tens of nanometers up to approximately 300 nm in length. These particles are present throughout nearly the entire amorphized zone (~40 nm thick) of low-Ca, high-Fe pyroxene lamellae, while they are found only in the upper part (~35 nm) of the amorphous zone in high-Ca, low-Fe pyroxene lamellae. In the solar wind-damaged zone of ilmenite, the maximum depth of np-Fe reaches ~40 nm from the surface [27]. Shen et al. (2024) investigated a series of CE-5 impact glasses and found three distinct sizes of np-Fe particles: particles smaller than 5 nm, with an average of 3 nm (Snp-Fe); particles ranging from 12 to 122 nm, with an average of 31 nm (Lnp-Fe); and larger particles from 85 to 970 nm, with an average of 411 nm (ULnp-Fe) [93]. The np-Fe in Chang’E-5 lunar soil is predominantly in the 0-valent state, although some larger np-Fe particles are surrounded by an oxide layer containing Fe^2+^ and Fe^3+^.

The return of the Chang’E-5 samples has advanced our understanding of the Moon and provided fresh insights into np-Fe particle formation. Due to the Moon’s highly reducing environment, trivalent iron (Fe^3+^) would be expected to be absent; however, remote sensing has detected hematite in high-latitude lunar regions [80,94]. Additionally, previous studies have reported trivalent iron in magnetite within lunar breccia and Apollo 17 soil, with Fe^3+^/ΣFe ratios in Apollo soil samples ranging from 0 to 25% (ΣFe = Fe^2+^ + Fe^3+^) [12,95,96]. Electron energy-loss spectroscopy (EELS) analyses of Chang’E-5 soil have revealed np-Fe particles alongside Fe^3+^ within amorphous silicates, with high-resolution TEM imaging showing no core–shell structures—indicating that Fe^3+^ formation did not result from atmospheric oxidation on Earth [51,89,97,98]. Molecular dynamics simulations suggest that the disproportionation of ferrous ions can occur under low temperatures and high pressures, which correspond to the secondary impact environments that produce amorphous layers [51].

Li et al. found abundant np-Fe particles in the amorphous layers resulting from micrometeorite impacts. These particles exhibited a larger diameter (~10 nm) in the shallow layers and a smaller diameter (~3 nm) in the deeper layers. Typically, smaller np-Fe particles are found on the surface of the amorphous layer, which is formed by solar wind reduction or vapor-phase deposition, while larger np-Fe particles are located within the amorphous layer, formed by molten thermal reduction [27,53,93]. However, Li et al. observed the opposite in their findings. EELS analysis revealed the presence of Fe^3+^ ions in the np-Fe region within the microcrater. Combined with a thermodynamic analysis, Li et al. suggested that these np-Fe particles are formed through disproportionation reactions (3Fe^2+^ = 2Fe^3+^ + Fe^0^) due to low-velocity impacts. Xian et al. (2023) observed that micrometeoroid impacts induce a disproportionation reaction that produces substantial Fe^3+^ (average Fe^3+^/ΣFe > 0.4) and generates np-Fe particles, identifying disproportionation as a likely mechanism for np-Fe formation in impact glasses [34]. Furthermore, Xian et al. (2024) reported that np-Fe particles in Chang’E-5 pyroxene fragments, formed through disproportionation, are larger than those generated by solar wind irradiation [100]. Low-velocity impact events can result not only from micrometeoroids traveling at low velocities but also from debris generated by high-velocity impacts. This suggests that low-velocity impacts occur much more frequently than high-velocity impacts. Therefore, np-Fe produced by impact-driven disproportionation reactions may be more widely distributed on the surfaces of airless bodies compared to np-Fe formed by evaporative deposition or solar wind exposure. This implies that larger np-Fe particles (>10 nm) on the surface of the amorphous layer are likely all formed by disproportionation reactions. Researchers have also observed rapid electron beam-induced oxidation (<200 ms) during TEM characterization of lunar impact glasses [101]. The presence and formation pathways of trivalent iron in lunar samples thus remain an open question, warranting further experimental investigation.

Recent findings from the Chang’E-5 mission have provided direct evidence of the decomposition of iron-bearing olivine into np-Fe particles in lunar soil samples. This process may play a crucial role in forming nanoscale metallic iron during the early stages of lunar soil development. Guo et al. (2022a), utilizing transmission electron microscopy (TEM) and electron energy-loss spectroscopy (EELS), characterized the microstructure of olivine edges in the Chang’E-5 samples and observed the coexistence of np-Fe with silica-rich materials [102]. These np-Fe particles appeared on the surfaces of the particles, with a particle size of approximately 35 nm. This large np-Fe particle was not formed by vapor deposition. In the rims of olivine grains, TEM analysis identified vesicular np-Fe associated with Si-rich material and an Mg-rich layer, providing a material basis for the formation of np-Fe. These microstructural features suggest that np-Fe particles may arise from the decomposition of sub-solidus olivine (Fe_2_SiO_4_ = 2Fe + SiO_2_ + O_2_) due to meteorite impact-induced fragmentation or localized heating processes. Additionally, Guo et al. (2022b) identified oxygen-bearing iron sulfide particles in the Chang’E-5 samples, which included magnetite and pure metallic iron [99]. This mineral assemblage indicates that FeO eutectic reactions (4FeO = Fe_3_O_4_ + Fe^0^) likely occur during magnetite formation (Figure 4b). Cao et al. (2024) observed a similar phenomenon, explaining that during the annealing process following micrometeorite impacts, a eutectoid reaction occurs within the interior of O-bearing iron sulfide grains at temperatures below 600 °C, forming a mineral assemblage of np-Fe, magnetite, and iron sulfide phases [103]. This process could facilitate the widespread presence of sub-micrometer magnetite in lunar soil and contribute to the iron content on the lunar surface. Furthermore, Zeng et al. (2024) discovered new space weathering products, trigonal Ti_2_O and triclinic Ti_2_O, which also accompanied the formation of np-Fe (3FeTiO_3_ = 3Fe^0^ + Ti_2_O + TiO_2_ + O_2_) [104]. Studies of Chang’e 5 lunar soil have revealed several new mechanisms for np-Fe particle formation, particularly on Fe-containing host particles (Table 1). This suggests that np-Fe particle formation is not the result of a single reaction, but rather a series of reactions induced by solar wind and micrometeorite impacts.

Guo, J. G. et al. (2022) were the first to report embedded wüstite nanoparticles (FeO particles) in lunar soil, which may act as intermediates in the formation of np-Fe particles [105]. These wüstite np-FeO particles, measuring 3–12 nm, were found within the amorphous layer of olivine, where np-Fe particles were absent, and no solar flare tracks were observed. In contrast, np-Fe particles were detected in olivine from the Chang’E-5 lunar soil, located within the amorphous silicate layer and exhibiting clear solar flare tracks. This comparison suggests that lunar soil samples from different locations have experienced varying degrees of space weathering. In addition to glass, np-Fe particles were identified in other weathered materials, such as mineral fragments, aggregates, and rock fragments, which have been reported less frequently in Apollo and Luna samples [27,34]. Lastly, Yan et al. (2024) observed multiple lunar soil samples, noting that metallic iron particles on the surfaces of weathered materials (excluding glass) may have detached from impact glasses and reattached to these surfaces [49].

The formation of np-Fe particles in lunar soil arises not only from the space weathering of surface materials but also from incoming meteorites [54]. In our solar system, it is estimated that over 90% of micrometeorites originate from the Jupiter family of comets and long-period comets [106]. These micrometeorites are primarily composed of iron (Fe), silicon (Si), and calcium (Ca), and they also contain various volatiles [107,108,109]. Upon impacting the lunar surface, the heat generated can cause the iron in these meteorites to form np-Fe particles through thermal reduction or vapor deposition. In cases where the meteorite’s impact velocity is low, the volatiles on its surface may be retained within the impact crater.

## 4. Simulation Experiments of Space Weathering

### 4.1. Simulation of Micrometeorite Impacts

Micrometeorite impacts are a significant factor contributing to space weathering on airless bodies. These impacts heat lunar soil particles, causing them to melt and initiating chemical reactions that modify the soil’s mineral composition. The instantaneous power density of pulsed lasers closely resembles that of typical micrometeorite impacts, and the laser spot size can be adjusted to match the diameter of an average micrometeorite impact (approximately 100 μm). Additionally, laser energy is distributed over a uniform area [110,111]. Consequently, pulsed lasers serve as a primary method for simulating micrometeorite impacts. Moroz et al. (1996) were the first to employ lasers for this purpose [112]. While their samples exhibited darkening and reddening, the pulse duration (0.5–1 μs) was too long compared to typical micrometeorite bombardment. Later, several researchers successfully utilized nanosecond pulsed lasers to simulate space weathering, more accurately reproducing the heating profiles of micrometeorite impacts and generating np-Fe particles with crystalline structures in the process [43,113].

**Figure 5:**
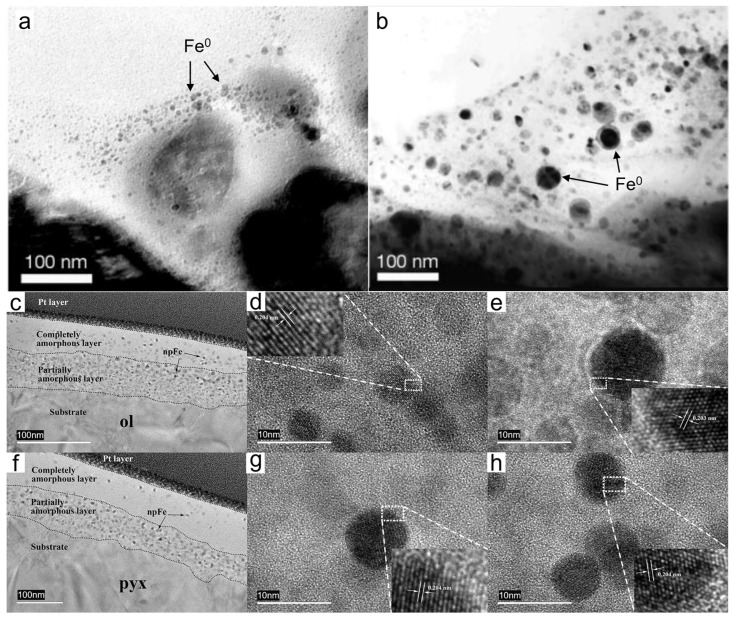
(**a**) Surface of olivine particles after 5 pulses of 30mJ pulsed laser irradiation, showing the presence of np-Fe particles [43]. (**b**) The surface of olivine particles after 20 pulses of 30mJ pulsed laser irradiation, with more and larger np-Fe particles. TEM of minerals in basalt after pulsed laser irradiation [43]. (**c**) TEM of olivine after pulsed laser irradiation, showing a three-layer structure. The black and gray circular spots in the amorphous layer represent np-Fe particles [31]. (**d**) HRTEM image of np-Fe in the completely amorphous layer in Figure 5c [31]. (**e**) HRTEM image of np-Fe in the partially amorphous layer in Figure 5c [31]. (**f**) TEM image of olivine after pulsed laser irradiation, showing a three-layer structure [31]. (**g**) HRTEM image of np-Fe in the completely amorphous layer in Figure 5f [31]. (**h**) HRTEM image of np-Fe in the partially amorphous layer in Figure 5f [31].

Initially, Hapke proposed that the laser ablation of lunar rocks resulted in a brownish deposit on the melted glass, which was thought to be caused by the formation of sub-micrometer Fe^0^ particles [2]. Sasaki irradiated olivine with pulsed lasers at a wavelength of 1064 nm and a duration of 6–8 ns, observing nanoscale iron particles at the edges of the olivine under transmission electron microscopy [43]. These np-Fe particles closely resemble those found in the rims of lunar soil grains in both their occurrence (with the surrounding amorphous material being richer in Si compared to the host mineral) and size (up to 30 nm) [8,53]. Notably, the absence of hydrogen in this simulation indicates that hydrogen implanted by solar wind is not essential for the formation of np-Fe particles in lunar soil (Figure 5a,b) [43]. This finding challenges the hydrogen reduction hypothesis and supports the vapor deposition hypothesis.

Sorokin simulated micrometeorite impacts on basalt using pulsed lasers and found that the impact craters displayed light and dark areas. The composition of the dark regions differed slightly from the initial glass, while the light regions were notably enriched in barely volatile oxides (Al_2_O_3_ and CaO) and depleted in volatile oxides (SiO_2_ and Na_2_O) [39]. Changes in the content of barely volatile and volatile elements align with the volatility series of petrogenic oxides (excluding FeO), suggesting that the light regions underwent evaporation differentiation. Two distinct distributions of np-Fe particles have been identified in both actual lunar soil samples and simulations. The first type, located at the edge of the crater, is generally interpreted to be formed by vapor deposition, while the second type, found within the volume of crater glass, is typically described as resulting from the thermal reduction of iron in the melt [3,17,39].

Micrometeorite impacts yield varying results due to the differing chemical compositions of minerals. The occurrence and morphology of np-Fe particles are closely related to the substrate’s composition [29]. Wu et al. (2017) irradiated basalt, feldspar, chondrite, and iron meteorites with pulsed lasers, revealing that the laser pits created by different rocks varied in shape and size: olivine and iron meteorites produced circular pits, whereas feldspar and chondrites exhibited irregular shapes (Figure 5c–h) [31]. Furthermore, Li et al. (2022) irradiated chondrites with pulsed lasers and discovered that np-Fe particles appeared in both olivine and pyroxene but not in plagioclase, likely due to its low iron content [33]. Additionally, the pulsed laser irradiation of magnetite did not result in the formation of np-Fe particles [66]. The outcomes of laser irradiation experiments depend not only on the mineral type but also on the iron content of the minerals. By irradiating simulated olivine samples with varying iron content (26.3%, 41.6%, 54.8%, 70.9%), it was observed that olivine with a lower iron content exhibited a vapor deposition layer and a melt glass layer, containing small, dense np-Fe particles. In contrast, olivine samples with a higher iron content lacked a vapor deposition layer, and the resulting np-Fe particles were larger and less abundant [32].

Through extensive simulation experiments, we can compare the effects of pulsed laser irradiation and ion irradiation on lunar minerals. Both types of irradiation induce similar spectral changes, leading to the darkening and reddening of samples across the visible to infrared spectrum [114,115]. However, pulsed laser irradiation has a more pronounced effect on spectral modification than ion irradiation. This suggests that micrometeorite impacts have a greater transformative influence on lunar soil compared to solar wind, although the stochastic nature of micrometeorite impacts means that their effects accumulate over time. Furthermore, while no ion irradiation experiments have produced np-Fe particles with crystalline structures, numerous pulsed laser experiments have successfully generated these particles. This further indicates that micrometeorite impacts play a more significant role in the formation of np-Fe particles. Pulsed laser simulations of micrometeorite impacts have also shown that the reddening and darkening of spectra are associated with the formation of np-Fe particles [25,30].

### 4.2. Simulation of Solar Wind Irradiation

The solar wind, primarily composed of hydrogen and helium ions (>99%), exhibits a broad velocity distribution with an average speed of 468 km/s (~1 keV/amu) [63]. Consequently, researchers often utilize H and He ions to simulate the solar wind and investigate its effects on the formation of np-Fe particles. Hapke (1973) first noted that the H^+^ irradiation of loose mineral powders, resembling lunar regolith, results in a visible darkening of the irradiated surface [40]. He attributed this effect to the formation of metallic iron in the sputtered vapor and its subsequent redeposition on nearby grains. Dukes (1999) irradiated olivine with 1 keV H^+^ and 4 keV He^+^ ions, and X-Ray photoelectron spectroscopy (XPS) analysis revealed that Fe^2+^ near the surface of olivine was reduced to Fe^0^, with the reduction being more efficient for He^+^ than for H^+^ [65]. Chaves et al. (2022) conducted similar experiments on magnetite, finding that 1 keV H^+^ and 4 keV He^+^ ions resulted in a decrease in oxygen content and an increase in iron content, with H^+^ ions having a more pronounced effect than He^+^ ions [66]. Low-flux H^+^ ion irradiation on meteorites dominated by olivine and pyroxene, with energy densities of 2 × 10^17^, 5 × 10^17^, and 1 × 10^18^ H^+^ cm^−2^, correspond to exposure times of approximately 93, 230, and 590 years at 1 AU, respectively. Significant spectral changes were observed, including a decrease in reflectance at 550 nm and reddening in the 1 μm region [28]. Loeffler et al. (2009) irradiated olivine with 4 keV He^+^ ions and determined through in situ chemical analysis that the reddening of the olivine spectrum after He^+^ irradiation directly correlates with the formation of metallic iron at the mineral’s surface, occurring within 50–80 Å from the surface [25].

While ion irradiation has demonstrated the ability to produce metallic iron through chemical analysis and spectral changes, the formation of np-Fe structures following irradiation has not yet been observed. Furthermore, the observed changes in spectral reflectance cannot be solely attributed to the formation of np-Fe particles [25]. As a result, the role of the solar wind in promoting the formation of np-Fe particles remains a topic of debate. When Murchison (CM2) carbonaceous chondrite was irradiated with H and He ions, a decrease in the carbon content was noted. Through transmission electron microscopy (TEM) analysis, Laczniak et al. (2021) suggested that low-flux H^+^ and He^+^ irradiation may not effectively reduce the natural Fe^3+^ and Fe^2+^ to Fe^0^ [26]. Numerous simulation experiments have been conducted to mimic the solar wind, employing H, He, C, and Ar ions at various energy densities [25,26,28]. The spectral changes observed after ion irradiation are comparable to those after pulsed laser irradiation; however, TEM analysis has revealed the presence of np-Fe particles following the pulsed laser irradiation of various minerals [31,114,116].

Moreover, ion irradiation experiments have demonstrated that the solar wind can modify the surface morphology of minerals. Typically, irradiation with H and He ions results in the formation of amorphous layers, vesicles, and cavities on mineral grains, which aligns with our observations of lunar soil [117,118]. Different mineral compositions display varying sensitivities to the solar wind. For instance, McFadden et al. (2022) studied lunar soil samples from Apollo 17 and determined that the maximum solar wind damage depth was 66 nm for plagioclase and 112 nm for olivine [119].

## 5. Conclusions and Prospects

This review summarizes the various formation mechanisms of np-Fe particles based on Apollo, Luna, and Chang’e lunar soil samples. Over the past few decades, extensive research has focused on lunar samples from the Apollo and Luna missions, as well as simulations of micrometeorite impacts and solar wind implantation. However, earlier studies were limited by inadequate equipment and microscale characterization techniques, leading to a heavy reliance on theoretical speculation. Based on the available data, researchers proposed three primary formation mechanisms for np-Fe particles: in situ thermal reduction of molten iron caused by micrometeorite impacts, vapor deposition resulting from these impacts, and hydrogen reduction due to solar wind irradiation. With the return of lunar samples from the Chang’E-5 mission and recent advancements in electron optics, new mechanisms have been identified, such as disproportionation reactions, eutectic reactions, and decomposition reactions. As research progresses, additional mechanisms may be uncovered.

Although many formation mechanisms of np-Fe particles have been identified, a definitive conclusion has yet to be reached. Space weathering on the Moon is a process that occurs over billions of years, making direct observation impossible. The mechanisms currently identified are not absolute; for example, np-Fe formed through disproportionation reactions has so far been observed only on mineral surfaces, and the hydrogen injected by the solar wind may also contribute to this process. As a result, many researchers have attempted to clarify the space weathering process through simulation experiments. In the past, pulsed laser irradiation has been widely employed to simulate micrometeorite impacts due to its short irradiation time and small interaction area, effectively mimicking the effects of these impacts without causing the mechanical damage associated with actual impact events [56]. In contrast, simulating micrometeorite impacts through heating typically involves using muffle furnaces or the microwave heating of bulk samples, which fail to replicate the unique effects of micrometeorite impacts [5,120]. Furthermore, previous experiments simulating solar wind and micrometeorite effects were largely non-in situ; samples were characterized before and after the experiments, lacking the real-time observation of chemical and microstructural changes during the experiments.

**Figure 6:**
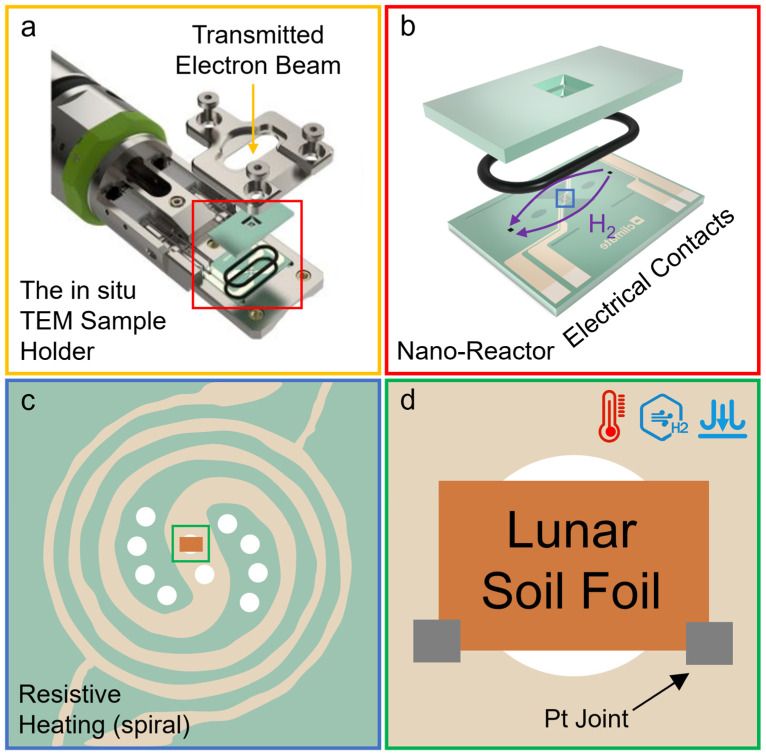
A Climate TEM holder for in situ gas and heating experiments. (**a**) Parts of a Climate TEM holder. (**b**) A nano-reactor consisting of a nanochip with the gas inlet and outlet, an O-ring for gas sealing, four electrical probes, and a top nanochip to enclose the reactor. The purple arrows indicate the gas inlet and outlet positions on the chip. (**c**) Overview of the resistive heating (spiral); the white circles are holes for TEM observation. (**d**) Lunar soil slices were soldered with Pt to the observational hole of the heated electrode.

Recent advancements in FIB-heating nanochip-TEM technology now enable researchers to simulate space weathering within the transmission electron microscope (TEM) and observe sample changes in real time (Figure 6).

Some scholars have conducted in situ heating experiments simulating micrometeorite impacts, successfully generating np-Fe particles analogous to those found in lunar soil. For instance, in situ heating experiments on Apollo lunar soil indicated that np-Fe particles form at approximately 575 °C, with their size increasing with subsequent heating cycles [46]. Howe et al. (2018) performed in situ heating experiments on carbonaceous chondrites, revealing significant alterations at temperatures exceeding 600 °C; after incubation at 800 °C for two minutes, np-Fe particles formed on the surfaces of certain silicate-containing particles [121]. In contrast to traditional simulated space weathering experiments, in situ experiments require a smaller sample volume, allowing for more efficient use of precious lunar soil. Additionally, in situ experiments can subject samples to thermal shocks of approximately 1000 °C, effectively simulating micrometeorite impacts. These experiments also enable the real-time observation of chemical and structural changes in the samples. These advantages make in situ experiments the optimal approach for studying the formation mechanisms of np-Fe particles and represent the future direction for simulating space weathering. However, the number of in situ heating experiments remains limited due to the high demands for micro-area instruments and specialized techniques. Furthermore, previous non-in situ simulated experiments have shown that the formation conditions of np-Fe particles can vary among different minerals. Therefore, to better understand the formation mechanisms of np-Fe in lunar soil, additional in situ experiments are crucial. Our future research will focus on this aspect, as the origin of np-Fe particles in lunar soil is still debated. We expect that more robust results will emerge from observations conducted during in situ heating experiments.

**Figure 7:**
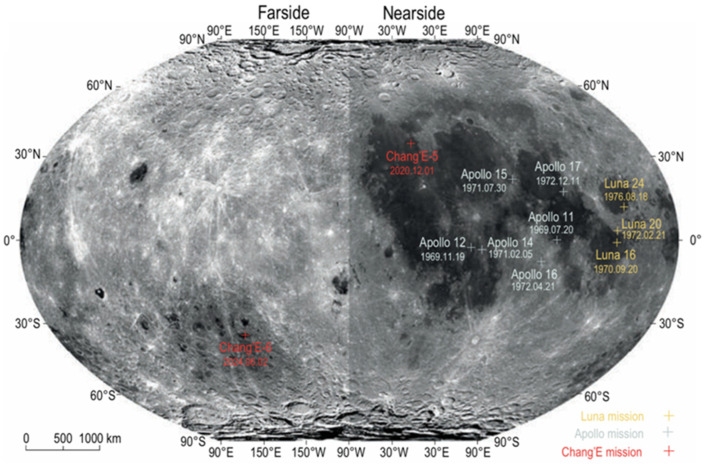
Distribution of lunar sampling sites and their collection dates [122].

Many countries, including the United States, the European Union, Russia, India, and Japan, in addition to China, have also announced plans for lunar exploration in the coming decades. The exploration of the Moon is regarded as a critical first step in humanity’s journey into space. The np-Fe particles in lunar soil can interfere with remote sensing spectra. Studying them allows for the correction of spectral data, leading to a more accurate mineral composition analysis. Additionally, np-Fe particles serve as important indicators of lunar soil exposure time, with a higher np-Fe content reflecting longer exposure and greater maturity. These particles are also found on other airless bodies similar to the Moon. Therefore, studying np-Fe in lunar soil not only enhances our understanding of the Moon but also informs the exploration of other extraterrestrial bodies. This year, the Chang’E-6 (CE-6) mission successfully returned the first samples from the unique South Pole–Aitken (SPA) basin on the lunar far side. The Chang’E-6 samples have, for the first time, revealed that young volcanic activity still existed on the far side of the Moon around 2.8 billion years ago, filling the gap in the record of lunar basalt samples from this period. The bulk chemical composition of the CE-6 samples indicates a lower degree of disaggregation and weathering of the original bedrock, with exogenous material incorporated during the regolith development of the basaltic bedrock at the CE-6 landing site (Figure 7) [122,123,124]. 

These samples will enhance our understanding of the Moon’s early evolution, as well as the composition and structure of its crust and mantle. The insights gained from CE-6 are expected to lead to new concepts and theories regarding the origin and evolution of the Moon, refining its role as a reference for understanding the evolution of terrestrial planets. Furthermore, these samples are anticipated to exhibit unique space weathering characteristics, offering new perspectives on the formation mechanisms of np-Fe in lunar soil.

## Figures and Tables

**Table 1:** Formation mechanisms and sources of np-Fe particles in space-weathered lunar sample.

Mechanisms	Reaction Formula	Characteristics of np-Fe Particles
Thermal reduction induced by impacts [38,39,46,47]	2FeO → 2Fe^0^ + O_2_	Particle size > 5 nm, small particles converge into large particles during the formation process
Impact vaporization and deposition [2,41]	2FeO → 2Fe^0^ + O_2_	Particle size is <10 nm and occurs in vapor deposition layers that are distinct from the host component
Solar wind reduction [42]	FeO + 2H → Fe^0^ + H_2_O	Particle size > 5 nm and accompanied by H_2_O
Impact-driven disproportionation [34,51,100]	3Fe^2+^ = 2Fe^3+^ + Fe^0^	Particle size ~10 nm and accompanied by the occurrence of Fe^3+^-bearing minerals
Sub-solidus decomposition of olivine [102]	Fe_2_SiO_4_ = 2Fe^0^ + SiO_2_ + O_2_	Particle size ~10 nm, occurs on the surface of olivine
FeO eutectoid reaction of magnetite [99]	4FeO = Fe_3_O_4_ + Fe^0^	Particle size > 30 nm, usually accompanied by meteoric iron sulfide and magnetite
Decomposition of ilmenite [104]	3FeTiO_3_ = 3Fe^0^ + Ti_2_O + TiO_2_ + O_2_	Particle size > 10 nm, accompanied by Ti_2_O appearing on the surface of ilmenite

## Data Availability

No new data were created or analyzed in this study. Data sharing is not applicable to this article.
